# Data-derived metrics describing the behaviour of field-based citizen scientists provide insights for project design and modelling bias

**DOI:** 10.1038/s41598-020-67658-3

**Published:** 2020-07-03

**Authors:** Tom August, Richard Fox, David B. Roy, Michael J. O. Pocock

**Affiliations:** 1 Centre for Ecology & Hydrology, Benson Lane, Crowmarsh Gifford, Wallingford, Oxfordshire UK; 2 Butterfly Conservation, Manor Yard, East Lulworth, Wareham, Dorset UK

**Keywords:** Data mining, Statistical methods, Biodiversity, Macroecology

## Abstract

Around the world volunteers and non-professionals collect data as part of environmental citizen science projects, collecting wildlife observations, measures of water quality and much more. However, where projects allow flexibility in how, where, and when data are collected there will be variation in the behaviour of participants which results in biases in the datasets collected. We develop a method to quantify this behavioural variation, describing the key drivers and providing a tool to account for biases in models that use these data. We used a suite of metrics to describe the temporal and spatial behaviour of participants, as well as variation in the data they collected. These were applied to 5,268 users of the iRecord Butterflies mobile phone app, a multi-species environmental citizen science project. In contrast to previous studies, after removing transient participants (those active on few days and who contribute few records), we do not find evidence of clustering of participants; instead, participants fall along four continuous axes that describe variation in participants’ behaviour: recording intensity, spatial extent, recording potential and rarity recording. Our results support a move away from labelling participants as belonging to one behavioural group or another in favour of placing them along axes of participant behaviour that better represent the continuous variation between individuals. Understanding participant behaviour could support better use of the data, by accounting for biases in the data collection process.

## Introduction

Human activities are causing changes in biodiversity at both global and local scales^1^. Climate change, land use change, and globalisation have led to wide scale loss of biodiversity^2^ and the translocation of species around the globe^3^, with negative impacts on ecosystem functioning^4,5^. Citizen science offers a mechanism to monitor the status and change of biodiversity, identify drivers of change, and report on progress in reversing declines^6^. Citizen science engages volunteers and non-professionals in scientific research^7^ and has grown and diversified rapidly in recent years, due in part to technological advances^8,9^. It provides benefits both in the engagement of people and in the cost-effective collection of environmental data^10^ and so offers a mechanism for large scale and long term data collection that would not be practical otherwise. Citizen science data are increasingly used to understand large scale processes in ecological and conservation biology such as the impacts of climate change^11,12^, pesticides^13^, invasive species^14,15^, and poaching^16^.

The diversity of citizen science projects is exemplified by projects that focus on the recording of wildlife observations. These projects range from highly structured recording schemes that require taxonomic expertise to participate, to simple opportunistic projects that encourage mass participation^17–19^. Allowing flexibility in the data collection process allows participants to choose how they engage with a project. This has been used to increase the engagement from participants^20^ but can result in biases in space, time and detectability that are larger than in structured surveys. Studies have explored biases in opportunistic data collected by citizen scientists^17,21,22^ and proposed statistical approaches for addressing these biases^23–25^. A number of recent studies have examined the use of data on individual participants to account for biases. These studies typically collect metadata at the time of the observations that include measures of expertise of the observer^26–29^, measures of effort^26,28,30–33^ (e.g. duration of the survey) and survey completeness^26,28,30,32–34^ (i.e. did the participant recorded every species they saw?). In some studies these variables are used to filter the data in an effort to remove biases that will hide signals of interest in the data. Survey completeness is commonly used to only include data where absences can be inferred because all species observed—within the taxonomic group of interest—were recorded^26,30,32^. It has also been shown that filtering data by a measure of effort can improve species distribution models^26^. As an addition or alternative to filtering, these metadata can be included in models to account for biases. Expertise and effort metrics have been used as covariates in occupancy models^27,29^, species distribution models^29^, mixed effects models^30,33^, logistic regressions^32^, and generalised additive models (GAMs)^31^ where they have been found to improve model performance. All these approaches require metadata to be collected by participants during the collection process, so are applicable to only a minority of citizen science projects where these metadata are collected. It is notable that most of the cited examples relate to the use of data from birdwatchers in North America, for whom there is a history of recording effort. We therefore lack metrics of recorder behaviour that can similarly be used to improve model outputs, but which are applicable to citizen science projects that do not capture so much metadata at the time of observation.

Previous studies have developed a number of metrics for quantifying the temporal variation in participant behaviour. Ponciano and Brasilerio^35^ used the framework proposed by O’Brien and Toms^36^ to create five metrics that describe the ‘engagement profile’ of participants in online citizen science projects, namely ‘Galaxy Zoo’ and ‘The Milky Way Project’. These metrics describing temporal patterns of engagement were used to define discrete groups of participants (‘profiles’). Boakes et al.^37^ adopted three of these metrics in their study of three biological recording datasets. The authors identified three ‘profiles’ of participants in their data, broadly representing “dabblers”, “enthusiasts” and those in between. The metrics used in these studies only address temporal variation in behaviour, however time is only one dimension in which participants can vary their data contribution. Where sampling protocols for field-based citizen science are more flexible, participants also vary in their spatial pattern of data collection and in the type of data they collect.

Given that un-accounted for variation in participant behaviour can create biases in the modelled outputs from citizen science datasets, it is valuable to quantify these types of variation in recording behaviour and their prevalence. The observed participant’s behaviours will vary from one citizen science project to another, leading to variation in the type and magnitude of bias in data between different projects. Variation in participant behaviour also creates positive opportunities for project organisers. Understanding the behavioural patterns of participants would enable project organisers to better design and tailor activities to their participants, and could help link known motivations^38,39^ to data quantity and quality. There is therefore a need for a common set of metrics that can be used to quantify variation in recording behaviour.

At present there are limited methods for quantifying variation across behaviours seen in field-based participants of citizen science projects. In this study we developed metrics to assess variation in the spatial pattern of observations and in data content, specifically the assemblage of species recorded in a multi-species citizen science recording projects. We compiled these with metrics assessing temporal variation, extending the work of previous studies^37,40^. We applied these metrics to a smartphone-based citizen science recording project in the United Kingdom (UK); ‘iRecord Butterflies’. We then tested the hypothesis that individuals can be categorised according to their observed behaviour^35,37^, and suggest how this can be used to address biases in the data and improve project design.

## Methods

Species observation data are defined by ‘what’ is reported, and ‘where’ and ‘when’ it is reported. Using these attributes we created three sets of metrics that describe variation in recording behaviour according to temporal distribution, spatial distribution and data content.

We applied our metrics to species sightings data from the iRecord Butterflies app (https://butterfly-conservation.org/8803/irecord-butterflies.html) from January 2014 to September 2017. This enabled us to understand the variation in participants’ behaviour from a single exemplar citizen science dataset. The iRecord Butterflies app was developed by Butterfly Conservation, the UK Centre for Ecology & Hydrology and Natural Apptitude (www.natural-apptitude.co.uk) and launched for free download to smartphones in March 2014 in both iOS and Android formats. The app provides one route for the submission of opportunistic butterfly sightings into the long-standing UK butterfly recording scheme, ‘Butterflies for the New Millennium’ (BNM) operated by Butterfly Conservation (www.butterfly-conservation.org). Sightings can be from any UK location on any date, present or past, and there is no sampling protocol. The app is designed to cater for a wide range of users from beginners to experts. It contains a species guide to all resident and immigrant butterflies native to the UK, including images, range maps, and information about ecology, phenology and identification. To submit sightings users must first create a free account in the iRecord online biological recording system developed by the UK Centre for Ecology & Hydrology. This allows us to reliably assign a unique user identity to all data recorded with the app. When using the app to create a butterfly record, users enter the one or more species observed, the number of individuals seen and the location name. The date and geospatial reference (latitude and longitude) of the sighting are added to the record automatically by the app, derived from the device’s clock and GPS, but can be manually changed by the user prior to submission. The user also has the option to attach an image of the species to the record.

The temporal and spatial metrics we present describe variation in the pattern of an individual's recording across time and space, while data content describes the variation in the assemblage of species they recorded. Metrics were grouped in this way to ease the interpretation of results and because we did not have an a priori reason to believe that the behaviours in one group (temporal, spatial, or data content) were dependent on any other group.

Some of the metrics we introduce cannot be reliably calculated for participants who have submitted only a small number of records. The number of active days that are required to get an accurate estimate of these metrics will depend on a number of factors including the number of taxa in to group being recorded, and variation in behaviour over time. An active day is defined as a day on which a reported butterfly was observed, even though the report may be submitted on a different day. Based on observation of the distribution of metrics and a sensitivity analysis (supplementary material), we selected 10 active days as our threshold. Participants below this threshold broadly match the ‘dabblers’ group identified by Boakes et al.^37^ both in terms of species observations made per individual (6.1), and proportion of participants allocated to this group (84%). We therefore started our analysis by removing all participants of iRecord Butterflies who were active on 10 or fewer days over the 4 years covered by the dataset.

The metrics we have used are designed to be as generalisable as possible and are broadly independent of the number of species in the taxonomic group being studied. The code used to create these metrics is available as an R package (github.com/BiologicalRecordsCentre/recorderMetrics). We encourage others to build on these metrics and submit contributions to GitHub for others to use.

### Temporal metrics

Temporal metrics were adapted from Ponciano and Brasileiro^35^ and Boakes et al.^37^. Given the seasonal nature of butterfly recording in the UK, we calculated the temporal metrics within only the summer periods, which were defined as the day range that contained the central 95% of the data for each year. Data from outside these summer periods was excluded from temporal metrics but included in spatial and data content metrics. If this is not done the temporal metrics are strongly correlated to the time of year a participant joined the project. The four temporal metrics we calculated were defined as:

- *Activity ratio* “The proportion of days on which the volunteer was active in relation to the total days he/she remained linked to the project”^35^. The time a participant is linked to the project is taken as the number of days between the first and last observation, once days outside of the summer periods are excluded.
- *Weekly activity* The median number of days a week the participant makes observations, when considering only the weeks in which the participant makes observations. This is an adaptation of the ‘Daily Devoted Time’ in Ponciano and Brasileiro^35^, but we use days in a week (summer periods only) rather than hours in a day. Unlike activity ratio this metric is not affected by periods of absence as it is only calculated across weeks when recording occurred.
- *Periodicity* The median number of days elapsed between each pair of sequential active days within a summer (i.e. not across years). This describes the regularity with which people record. Years with a single observation do not contribute information to this metric.
- *Periodicity variation* “The standard deviation of the times elapsed between each pair of sequential active days”^35^. In our analysis this is estimated for the summer period only (i.e. not across years).

We did not use the metric ‘Relative activity duration’, which was used by Ponciano and Brasileiro^35^. This metric is calculated as the number of days a participant was active divided by the days elapsed since the participant joined the project until the project finishes. Biological recording projects are typically open ended, indeed many already span multiple decades, making this metric less informative.

### Spatial metrics

**Figure 1:**
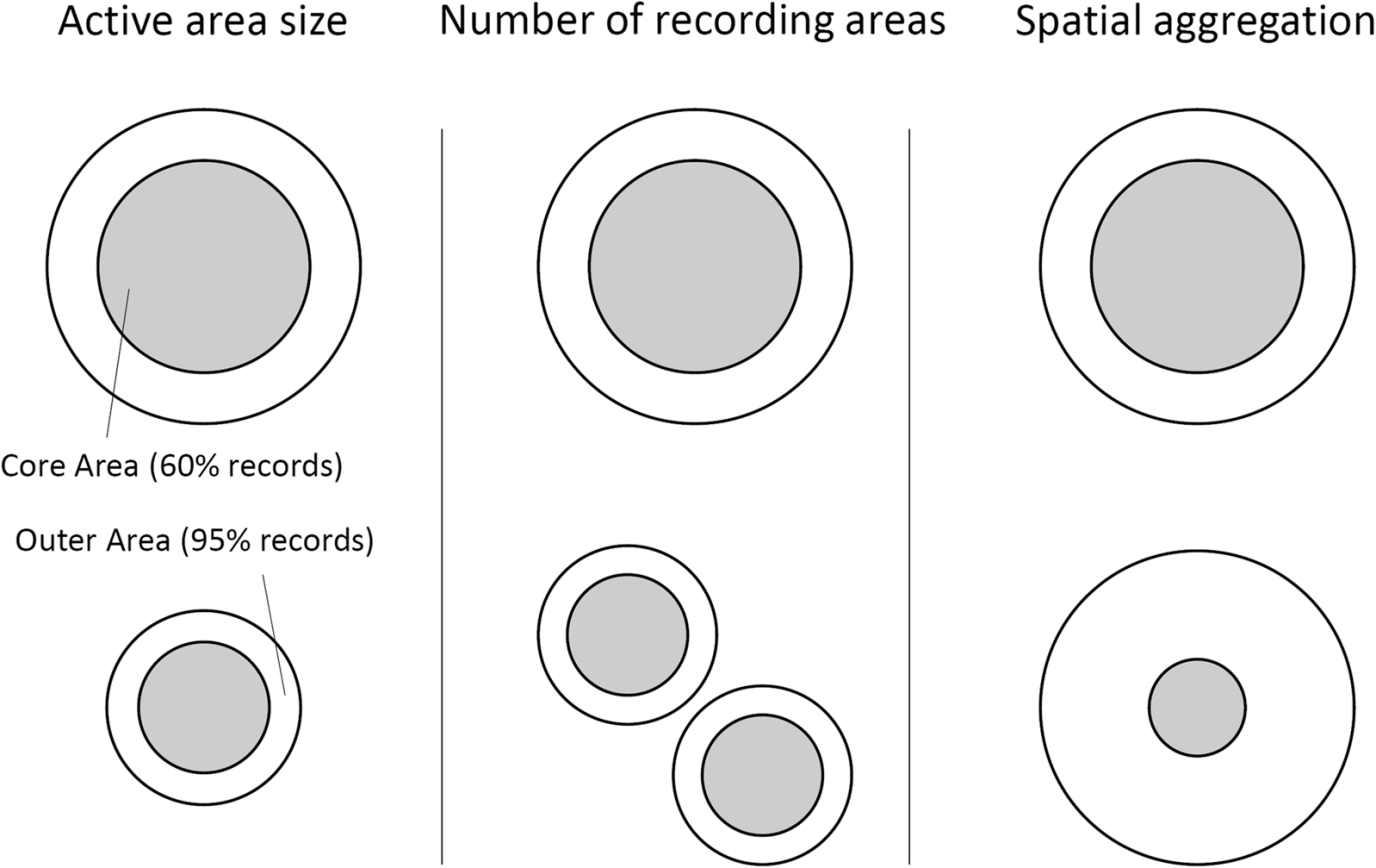
Illustration of spatial metrics. Each of the three spatial metrics are independent, the change from the top row to the bottom row represents a change in the given metric where the other two metric are held constant. The circles are illustrative; kernel density polygons can take any shape.

The set of spatial metrics were calculated across all records, regardless of whether they fell in the summer period or not (Fig. 1). The metrics were based on the kernel density polygon (or polygons) of the point records for each participant. A 95% kernel density polygon estimates the minimum area that has a 0.95 probability of containing the participant’s next record. Kernel density polygons were estimated using the R-package adehabitatHR^41^, and can be a single polygon or a multi-part polygon.

- *Active area size* This is the area of a 95% kernel density polygon fitted to a participant’s observations. This describes the spatial extent of the majority of a participant’s recording activity.
- *Number of recording areas* This is the count of the number of discrete, non-intersecting, polygons that make up the active area. Larger numbers indicate that a person records in many different locations. Smaller numbers indicates that a person records in fewer locations and/or more homogeneously across the landscape.
- *Spatial aggregation* This is the core recording area (the 60% kernel density polygon) as a proportion of the active area size (the 95% kernel density polygon). This gives an indication of how aggregated the data points are. A small value indicates records are concentrated in a relatively small area (or areas) with scattered records elsewhere, a large value indicates that records are distributed more homogeneously.

### Data content metrics

The set of data content metrics capture the variation in what participants record. All data collected by each participant is used to calculate these metrics:

- *Proportion of taxa recorded* This is the number of unique taxa recorded by an individual as a proportion of the total number of taxa recorded by all participants.
- *Rarity recording* Taxa are ranked according to the number of records in the entire dataset from highest to lowest and scaled to 100. The most-rarely reported species has the value 100 and the most-commonly reported a value of 1: this is the species’ rarity value. The Rarity Recording metric is the median rarity value, across all records for the participant, minus the median rarity value across all observations in the dataset. Negative values of this metric show that the participant submits records of common taxa more frequently than expected, while positive values mean that the participant submits records of rarer taxa more frequently than expected.
- *Single-species lists* The proportion of visits (unique combinations of date and 1 km square location) on which the participant submitted a single record. This gives the proportion of records that are ‘one-off’ observations of single species as opposed to those where a participant has submitted records of several species from the same location at the same time. A high value represents frequent ‘one-off’ records whereas low values indicate more comprehensive searching and reporting.

Some of these metrics were log_10_ transformed to remove skew (specifically: active area size, number of recording areas, periodicity variation, periodicity, and activity ratio). A small value of 0.001 was added to these variables prior to log_10_ transformation to prevent large negative values. All variables were included in a k-means clustering analysis to identify grouping of individuals in the data. All variables were scaled and normalised prior to clustering by centering on the mean and dividing by the standard deviation. Simple Structure Index was used to identify the optimal number of clusters^42^ with 5 set as the maximum number of clusters to consider. Average silhouette width was used to test for goodness-of-fit of clustering results. Values less than 0.5 were taken as evidence that the clusters were not well supported^43^. Where there was poor evidence for the presence of distinct clusters a principal components analysis (PCA) was used to reduce the dimensions of the metrics and identify the primary drivers of variation. The relationship between principal components derived from temporal, spatial, and data content metrics were assessed using linear regressions. All analyses were undertaken in R version 3.4.1^44^. Kernel density estimation was performed using adehabitatHR v0.4.15^41^, clustering was undertaken with vegan v2.5-1^45^, and cluster v2.0.7-1^46^, principal components analysis was performed in base R.

### Ethical approval

This research forms part of a programme of work to “inform the conservation of butterflies” in accordance with the app privacy policy. Further ethical approval was not required because names were anonymised, location data were aggregated and only aggregated metrics are analysed. The use of personal data complies with the EU General Data Protection Regulations; no personal sensitive information was obtained during this project and personal data were not shared outside of the research team.

### Informed consent

Informed consent for the use of personal data was received at the point of registration in the iRecord Butterflies app.

## Results

**Figure 2:**
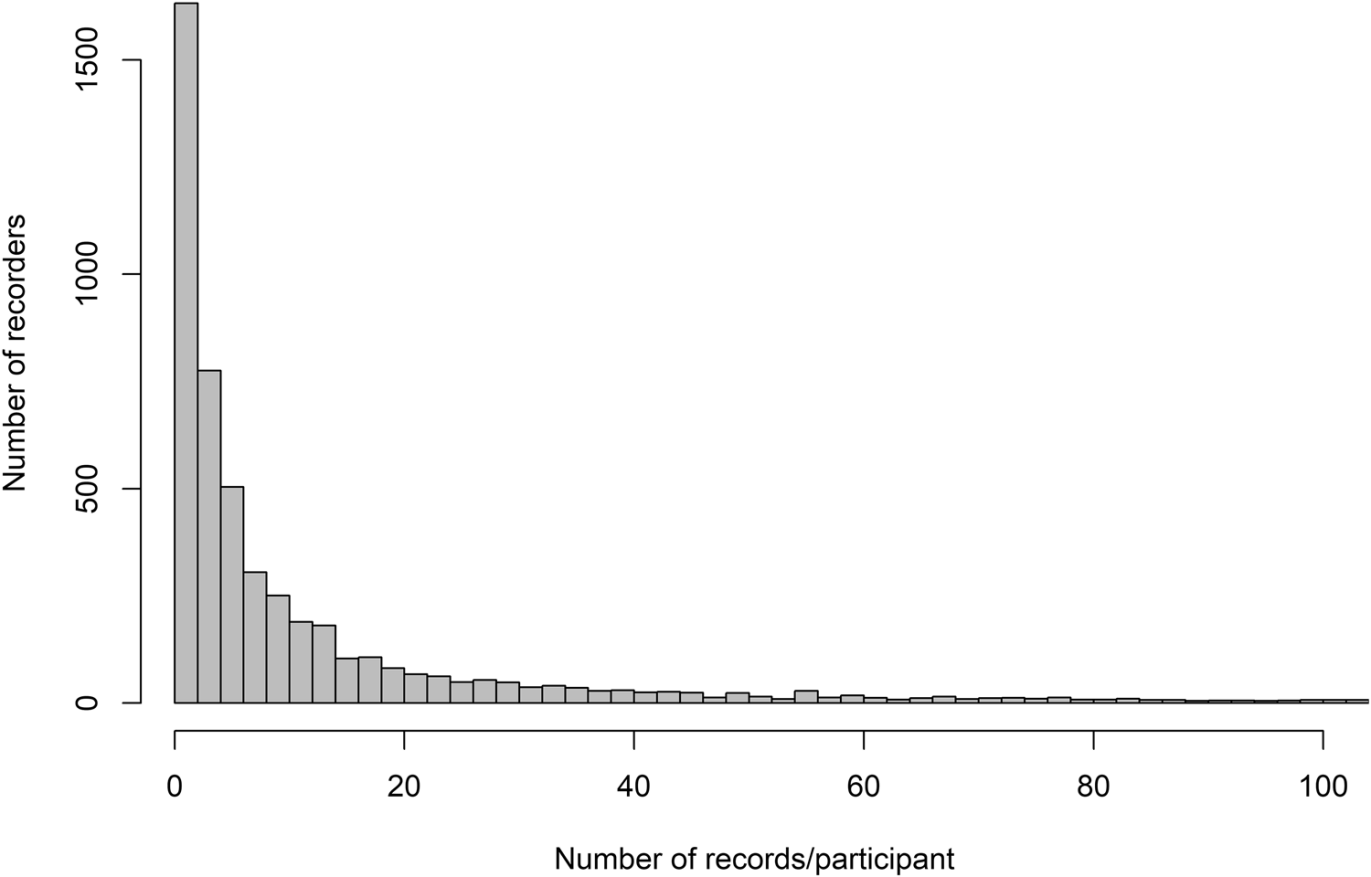
A histogram showing the distribution of records contributed per participant. The x-axis is truncated at 100. The top 14% of participants contribute 80% of the data.

Over the period January 2014 to September 2017, 169,707 records were submitted by 5,268 participants via the iRecord Butterflies smartphone app. 4,268 (81%) participants were removed from the analysis as they were active on 10 or fewer days, and participant behaviour metrics could not be reliably calculated. Specifically estimates for several of the metrics showed substantial bias or variation when there are fewer than 10 active days of data from which to make an estimate (supplementary material). This matches closely the proportion of ‘dabblers’ (84%), characterised by low levels of contribution, as reported by Boakes et al.^37^ for biological recorders using the iRecord submission tool in London. In our study these participants submitted an average of six records per person. The removal of these participants focuses this study on more engaged participants who contributed most of the records in the dataset. We tested the sensitivity of our results to this threshold, which can be specified by users of our R-package, and found no difference to the results when using a thresholds of 7, 10, or 15 (supplementary material). The participants who were active on > 10 days (1,000 participants in total) contributed 84% of all records to the project during the sampling period (Fig. 2), an average of 143 records (4,440 maximum) per person.

**Figure 3:**
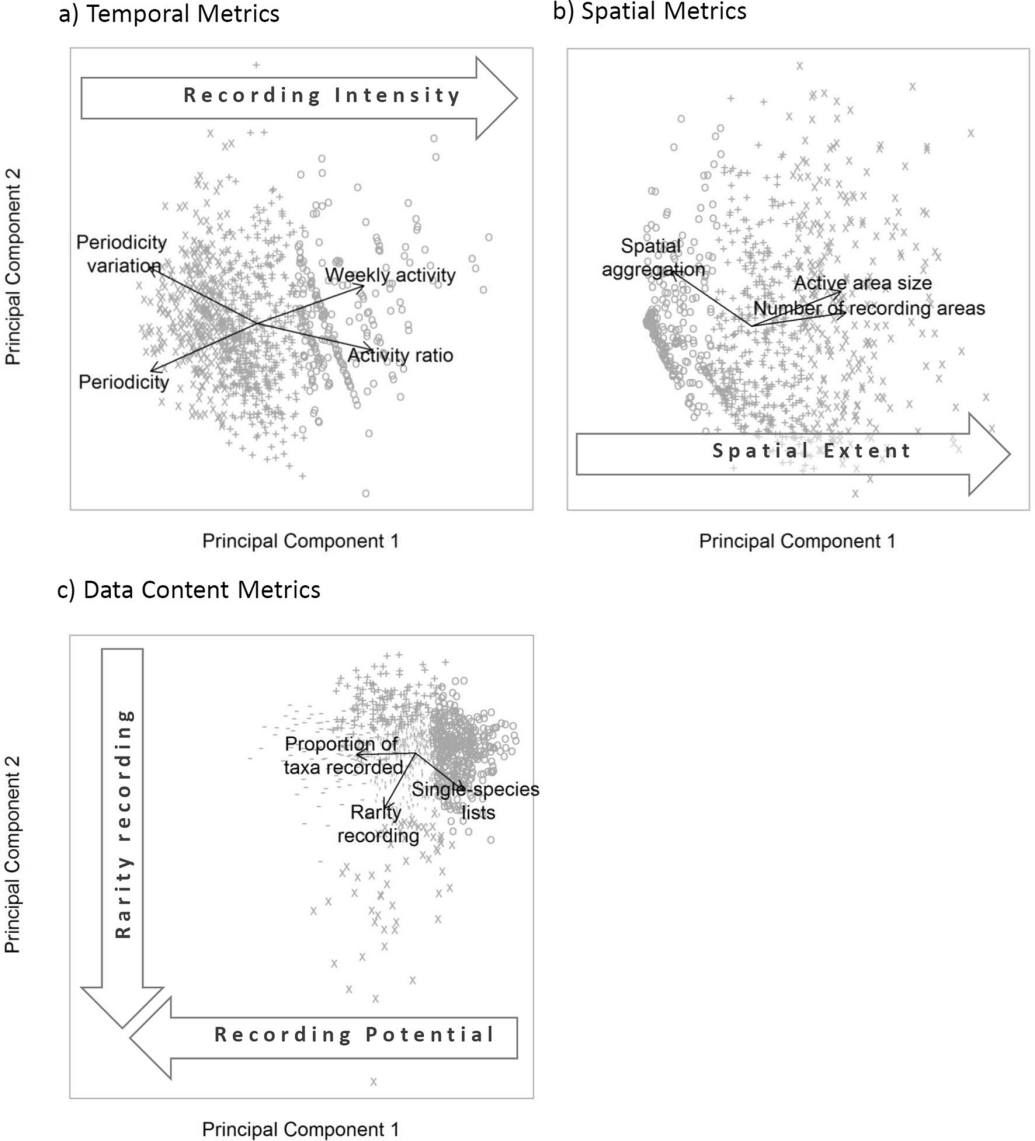
Principal components analysis undertaken using the three sets of participant metrics: temporal, spatial and data content. Symbols represent the clusters identified via k-means clustering, but which have low support across all three sets of metrics.

K-means clustering was used to cluster participants by their (1) temporal, and (2) spatial recording behaviour, and (3) the data content of their records. The Simple Structure Index suggested that the optimal number of clusters for temporal, spatial and data content metrics were 3, 3, and 5 respectively. However, the average silhouette width (0.33, 0.41 and 0.28) showed that clusters are poorly supported by the data^43^. Inspection of the relationships between clusters and participant metrics suggested that clusters fall along a continuum rather than representing discrete groupings of participants. This continuum was most apparent when the suggested clusters were plotted onto PCAs (Fig. 3).

Among temporal metrics the first principal component explained 74% of the observed variation (Fig. 3a). This axis describes recording intensity; individuals with high values record frequently, regularly and with low periodicity.

The first principal component of spatial components explains 82% of variation (Fig. 3b). This principal component describes spatial extent; participants with high spatial extend record over many locations. Participants with low spatial extent record locally and their records are more aggregated.

Data content metrics require two principal components to describe the majority of the observed variation (86%, Fig. 3c). The inverse of the first principal component (52% of the observed variation) represents recording potential. Participants with high values have recorded a large proportion of species, record fewer lists of length one on a site visit and record more rare species than the average participant. As a consequence, on an average visit to a site, these participants are likely to record more comprehensively than those with low recording potential. The inverse of the second component (34% of the observed variation) describes rarity recording. A participant with a high rarity recording score will tend to preferentially record rarer species, and have a greater propensity to record lists of length 1. So-called ‘twitchers’, i.e. those who specifically go looking for rare species, would have a high rarity recording score if they only reported the species they went looking for.

**Figure 4:**
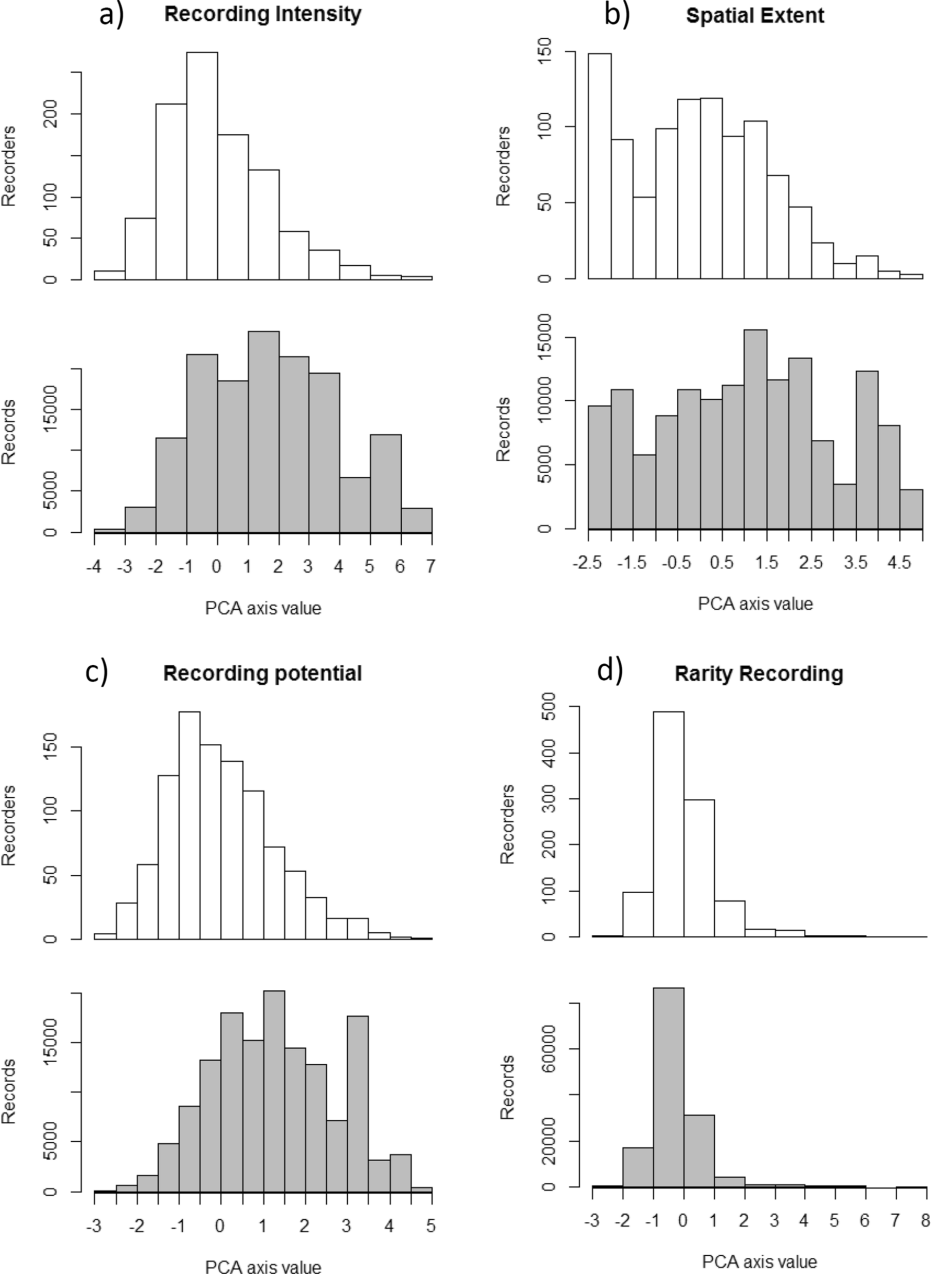
The distribution of participants and records along the 4 axes of participant behaviour. Upper (white) plots show the distribution of participants while lower (grey) plots show the distribution of records (i.e. the sum of records contributed by participants in each white column). Number of records per participant is significantly positively correlated to the axes of participant behaviour in (**a**–**c**), and negatively correlated to (**d**) (see Fig. 5).

The number of records contributed by participants depends on where they fall along each of the four axes of participant behaviour. To show this we first plot the distribution of participants along each axis of participant behaviour (Fig. 4—white columns), and second sum the number of records submitted by participants in each white column and plot this (Fig. 4—grey columns). Variation between these two plots arises because of correlations between the axes of participant behaviour and the number of records submitted per participant.

There is a skew in the distribution of participants along the recording intensity axis which shows a minority of participants record frequently, and regularly (Fig. 4a, upper) but contribute a large proportion of all the records (Fig. 4a, lower). The distribution of spatial extent suggests a bimodal distribution of participants along this axis (Fig. 4b, upper). The peak at the lower end represents participants who record in a single, small location. This is probably a distinct behaviour, e.g. those participants who record only in their garden or a local park. The distribution of participants along the recording potential axis is skewed to the lower end while the distribution of records is skewed in the opposite direction (Fig. 4c). This shows that participants with higher recording potential are contributing more data per individual. It should be noted that for participants to score a high recording potential they must have recorded many different taxa and few lists of length one, which is highly unlikely if they have made few records. There is a tail of participants with high values of rarity recording, indicating a small number of participants have a strong preference for recording rare species and single species lists, the distribution of records suggests these participants produce fewer records per individual.

**Figure 5:**
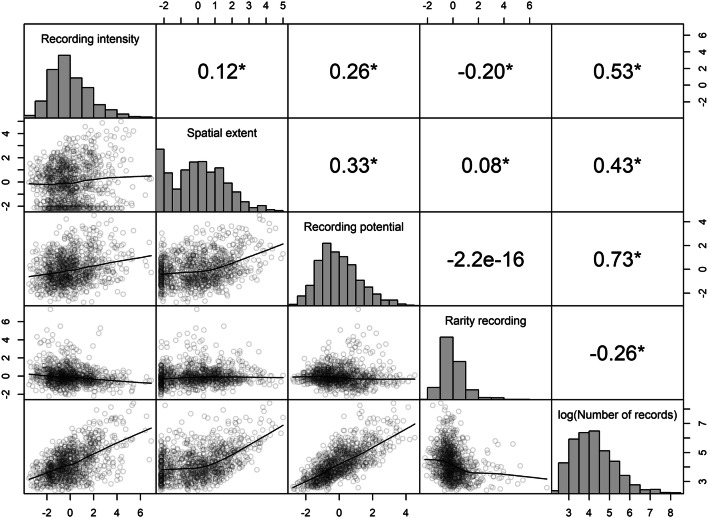
Correlation between the four axes of participant behaviour identified, and against log_10_ number of records per participant. There were significant associations (shown with ‘*’ where *p* < 0.05) between all variables except for rarity recording and recording potential. Numbers above the diagonal are Pearson's correlation coefficients. Below the diagonal the relationship is illustrated with a lowess line.

The axes of participant behaviour we describe are correlated to each other in the iRecord Butterflies dataset. Results from a linear regression showed that recording intensity, spatial extent and recording potential were all positively correlated with each other (Fig. 5). However, the correlation coefficients of these inter-axis relationships was relatively low (0.16–0.33) and suggests that it is not possible to accurately predict one axis value from another for any one participant. Rarity recording had a weak positive relationship with spatial extent, a negative relationship with recording intensity, and no association with recording potential (which was expected since they were orthogonal axes from the same PCA). The negative relationship with recording intensity supports the hypothesis that these participants are choosing not to record common species that they encounter.

Recording intensity, spatial extent and recording potential were all significantly positively correlated with the log_10_-transformed number of records per individual (*p* < 0.05), while rarity recording was significantly negatively correlated (*p* < 0.05). These relationships are relatively strong compared to the inter-axis correlations (Fig. 5). This supports the conclusion that participants who are active over a large spatial extent, record intensely, and record a lot on each site visit, are also the largest contributors of data. We also learn that those who prefer to record rare species make fewer records overall, this may be a result of their motivation against recording everything they see or because they record in areas with relatively few, but rare, species.

We found similar results when we undertook a PCA across all metrics (temporal, spatial, and data content combined) in which the first four principal components explained 82% of the variation. The first four principal components in the combined analysis can be mapped to the same four axes of participant behaviour identified here (supplementary material) however given the high dimensionality (10 metrics), the interpretation is not as clear-cut.

## Discussion

Participants in our multi-species citizen science study varied in their recording behaviour, in space, time and the type of data they recorded. The majority of participants recorded very few records with 81% of participants being active on 10 or fewer days, and contributing only 16% of the data. Boakes et al.^37^ term these participants ‘dabblers’ since their engagement in the project are typically short in duration and small in their overall contribution to the dataset. Our result matches reports made across numerous citizen science projects^17,47,48^ which show that the majority of data is produced by a minority of participants.

Previous studies have tried to place individual participants into clusters^17,35,37,40,49^, exhibiting one group of behaviours or another. These clusters inevitably vary between studies making comparison, and therefore generalisation across studies, difficult^35,40^. In contrast to previous studies once we removed participants active on 10 or fewer days we did not find significant evidence of clustering, and instead we found individuals’ behaviour fell along a continuum. So, while being able to label individuals as being one ‘type’ or another is appealing, we suggest it is misleading. Using continuous axes of participant behaviour is a more accurate description of the variation in participant behaviour. This also increases the potential of future studies to explain these behaviours (e.g. through qualitative assessment of motivations), or to detect changes in behaviour over time.

We identify four axes of participant behaviour representing recording intensity, spatial extent, recording potential and rarity recording, which explain the majority of variation observed in the three groups of metrics we assessed. The recording intensity, spatial extent and recording potential axes give a more detailed description of the uneven contribution of data across participants of citizen science. This provides insights into the behaviours that explain the skew, sometimes called the 80:20 rule or 90-9-1 rule^47,48^. We show that so called ‘super users’, those who contribute the most data^50,51^, are more likely to be recording intensely, over large areas, making more records than an average participant when they are in the field, and not focusing solely on rare species.

Projects and models should take account of participant behaviour in their design and implementation. Those that seek to explore intra-annual variation in phenomena should consider the recording intensity axis. Individuals with low values record infrequently and typically over a short time period which may not be sufficient for the project outcomes. The spatial extent axis reveals a bimodal distribution in spatial recording behaviour (Fig. 4b). There is a peak in participants who record in very small areas, which will result in pockets of high density recording in the data. These participants could be targeted for studies which required many repeat visits in the same location, for example to assess trends in species over time. The potential for a participant to make observations on an average outing is captured in the recording potential axis. This is driven by the participant’s experience in recording the taxonomic group (reflected in the proportion of all species they have recorded and their above average recording of rarities), and their preference for reporting lists of species rather than individual sightings. Participants with high values of recording potential are likely to be taxonomic experts, and will be well suited to projects where thorough site surveys are required. However, the recording potential axis is susceptible to spatial variation in species richness; two participants, otherwise identical, in areas of differing species richness will have differing recording potential values. It therefore captures attributes of the individual and the ecological and biogeographical context in which they typically record. Bias in the detectability of rare and common species at the level of the individual participant are revealed in the rarity recording metric. Participants with a preference for recording rarities might be particularly motivated by opportunities to record new and rare taxa such as invasive species. Conversely, those participants only recording common species may not have the skills required to identify rare species.

The metrics and axes we have developed can be used in data analyses to account for biases in data collection created by variation in recorder behaviour. Biases can be addressed by filtering data to a less-biased subset prior to analysis^26^, and our metrics and axes provide a range of options for this approach. Studies that rely on the assumption that non-detection indicates absence, such as occupancy models, could filter users based on the recording potential axis, thereby removing participants who tend to record short lists of common species, and who have recorded a small proportion of taxa overall. Alternatively the rarity recording metric can be used to reduce bias in detectability of either rare or common species, or both.

Beyond filtering, our metrics and axes can be used to account for biases in the data using models, a technique that has been found to improve the accuracy of model outputs^29,32^. Previous studies have used measures of expertise that are estimated using information such as survey duration^52^ as model covariates^29^. Our recording potential axes is broadly analogous to expertise but can be estimated without survey metadata such as duration. Recording potential could be included as a covariate in models including linear models, mixed effect models, species distribution models and occupancy models to account for variation in expertise. For example, as part of a hierarchical occupancy model, recording potential could be included in a detection sub-model to account for recorder level variation in detectability. Furthermore the rarity recording axis can be included as an interaction term with ranked species rarity to account for individuals who preferentially record common or rare species.

Where estimates of recorder behaviour metrics are not possible for a portion of participants, such as those active on 10 or fewer days, or where the participant identity is unknown, the metrics can still be included in Bayesian models, such as hierarchical occupancy models. In these cases the available estimates can be used and an uninformative prior can be used for individuals without recorder behaviour metric estimates, or without a known participant identity.

The distribution of participants along the four axes of participant behaviour we present will vary across field-based citizen science projects. Indeed, PCAs of the participant metrics presented here may well result in new axes of participant behaviour in future studies of different projects. The R-package we have created (https://github.com/BiologicalRecordsCentre/recorderMetrics) allows users to easily create the metrics presented here, which can be used in PCA’s to test for the key axes of participant behaviour in other citizen science projects. The axes of participant behaviour based on the PCA results from this study can also be calculated using *recorderMetrics*, which will allow a fair comparison across studies.

Results from future analyses using these methods will differ according to who is engaged, and how. We studied people interested in recording butterflies via a smartphone app. Comparisons of participant metrics between projects using different submission methods will be able to establish the affect this has on participant behaviour. Likewise, the sampling protocol used will also impact participant metrics. For example, a citizen science project that specifically asks participants to submit sightings from their garden (e.g. the Big Garden Birdwatch: https://ww2.rspb.org.uk/get-involved/activities/birdwatch) will have little variation in spatial metrics.

We encourage others to use, and build on, these methods. When using these metrics there are some common pitfalls to consider. For many of the metrics presented, a reliable estimate cannot be obtained if only a small number of observations have been made by an individual (we used 10 active days as the threshold). When calculating temporal metrics it is important to consider large scale patterns in temporal recording such as the seasonal variation in recording seen in iRecord Butterflies. Failure to account for this will mean that the metrics are biased by the time of year that a participant joins a scheme. The data content metrics we present assume that all individuals have the same opportunity to observe and record all taxa. This is the case in our study as long as individuals are willing and able to travel across the UK. However, for biodiversity recording, an individual may record all the species locally present, with additional species being regionally present. This will affect ‘Proportion of taxa recorded’ and ‘Rarity recording’ metrics. Therefore, large-scale projects will need to calculate these metrics at spatial scales that are relevant to the scale at which participants are likely to travel. We demonstrate how this can be achieved using either regional boundaries (e.g. country borders), or buffers around the data for each recorder in the documentation accompanying the *recorderMetrics* R package and show the impact of this approach on the iRecord butterflies metrics in supplementary material. The metrics require that each data point is accurately associated with a unique individual. This is feasible for many citizen science projects with electronic submission, but this is not always the case, either because the name was not recorded or because the name is not stored in an unambiguous format (e.g. ‘A. Smith’, ‘A.D. Smith’, ‘Antony Smith’) making it difficult or impossible to uniquely identify individuals.

Our participant metrics and axes of participant behaviour provide an opportunity to examine how participants’ behaviours change over the duration of their engagement with a project. iRecord Butterflies spans only a short period (4 years), during which we have assumed that individual participant behaviour is unlikely to change, however, future studies could examine changes in behaviour of participants over time, including the drivers of change which might have implications for the design of citizen science projects and engagements.

This study highlights both opportunities and challenges for practitioners of citizen science. Firstly, the behaviour of citizen scientists is highly variable, providing a challenge for models that use these data to account for this variation in their analyses. Analytical models for citizen science data to date have employed various methods to account for spatial, temporal and recorder effort bias, typically at the level of the overall dataset^23,24,53,54^. We recommend that our metrics could be used to account for individual-level patterns of recording, to reduce bias in the model outputs^29^.

Secondly, organisers of successful citizen science projects understand what motivates and interests participants and design their projects accordingly^39,55–57^. However, linking motivations to behaviours is being held back by the absence of a common set of metrics that can be used to investigate variation in participants’ behaviour. Understanding this link is key to designing citizen science projects that satisfy participants’ motivations and deliver data to answer research questions. We believe our metrics and axes of participant behaviour offer an important basis on which to build this research in the future.

## Supplementary information

Supplementary file 1 (CSV 177 kb)

Supplementary file 2 (PDF 1158 kb)

## Data Availability

The code to reproduce these analyses is openly available at https://github.com/BiologicalRecordsCentre/recorderMetrics. Full documentation for the package, including a tutorial, can be found at https://biologicalrecordscentre.github.io/recorderMetrics/. The version of recorderMetrics used in this paper can be found at https://github.com/BiologicalRecordsCentre/recorderMetrics/releases/tag/0.9. The participant metrics, as well as the values for each axis of participant behaviour, for all users of iRecord Butterflies are shared with this manuscript in an anonymised format (supplementary dataset file).
