# Supplementary material for: Data-derived metrics describing the behaviour of field-based citizen scientists provide insights for project design and modelling bias
Source: Sci Rep. 2020 Jul 3;10:11009. doi: 10.1038/s41598-020-67658-3 (PMC7334204; doi:10.1038/s41598-020-67658-3)
Supplement: Supplementary file 2 — (PDF 1158 kb) [file 41598_2020_67658_MOESM2_ESM.pdf]

Data-derived metrics describing the behaviour of field-based citizen scientists provide insights for project design and modelling bias

Tom August*[1], Richard Fox[2], David B. Roy[1], Michael J.O. Pocock[1]

[1] Centre for Ecology & Hydrology, Benson Lane, Crowmarsh Gifford, Wallingford, Oxfordshire, UK

[2] Butterfly Conservation, Manor Yard, East Lulworth, Wareham, Dorset, UK

* Corresponding author: tomaug@ceh.ac.uk

Supplementary Materials

Contents

1 – Effect of active day threshold 3
2 – Principal components analysis of all metrics in a single analysis 7
3 - Estimating recorder metrics for spatial subsets 9
Methods 9
By Country 9
By Buffer 13

1 – Effect of active day threshold

Some of the metrics we introduce cannot be reliably calculated for participants who have submitted only a small number of records. We therefore started our analysis by removing all participants of iRecord Butterflies who were active on 10 or fewer days over the four years covered by the dataset. An active day is defined as a day on which a reported butterfly was observed, even though the report may be submitted on a different day. How many active days are required to get an accurate estimate of these metrics will depend on a number of factors including the number of taxa in to group being recorded, and variation in behaviour over time. We selected 10 active days since broadly matches the 'dabblers' group identified by Boakes *et al* 2016 both in terms of observations made per individual (6.1), and proportion of participants allocated to this group (84%).

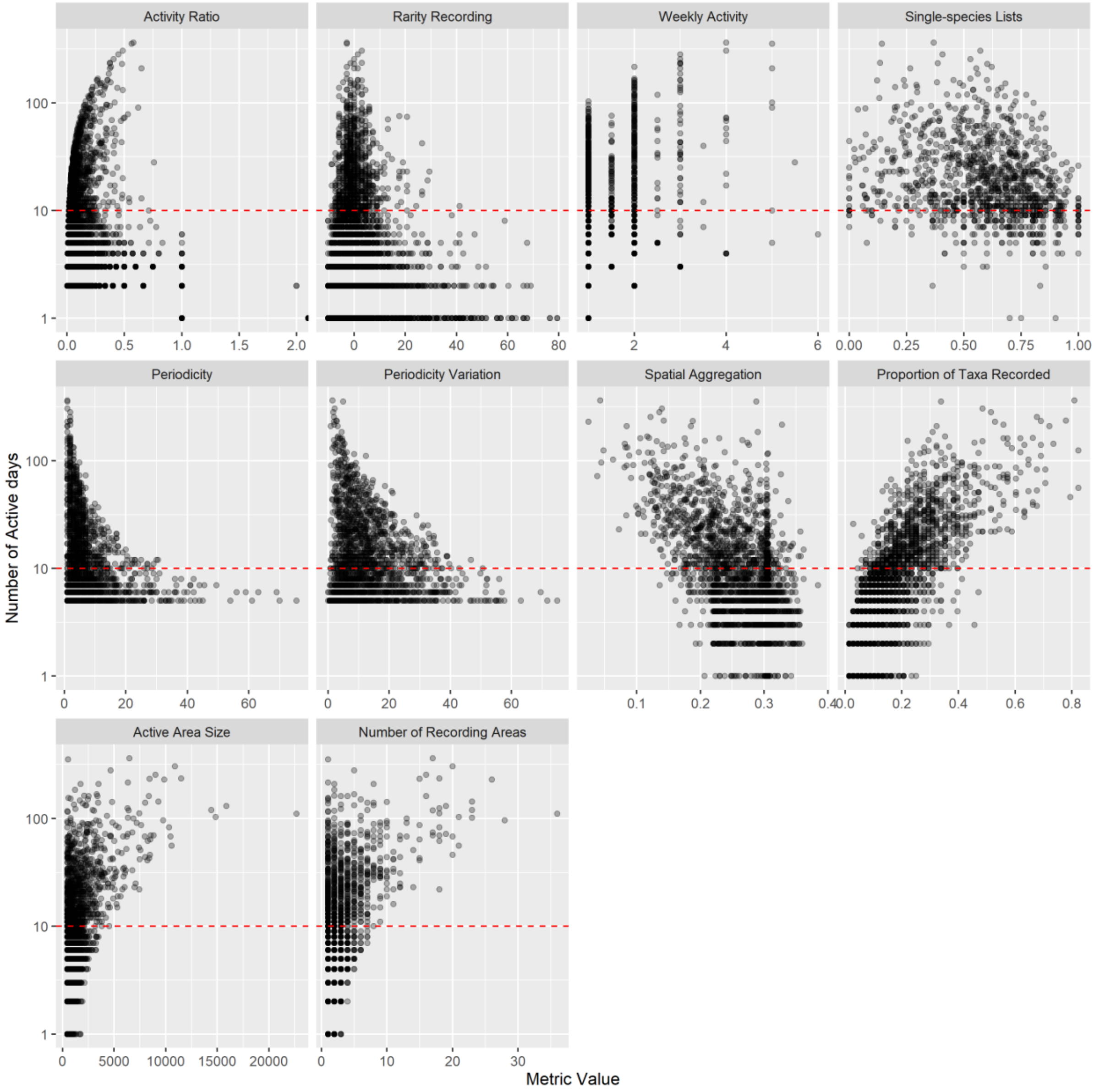

S1 – Recorder metric estimates plotted against the number of active days. The red dotted horizontal line indicates 10 active days. Periodicity and periodicity variation were not calculated for recorders with fewer than 5 active days. Note that the vertical axis is on the log scale.

Here we show what metrics look like for participants with fewer than 10 active days (figure S1). When the number of active days, and by definition number of observations, is low we see a large increase in the number of extreme values. This makes intuitive sense since the sample size for making estimates is small. As a result metrics that are attempting to average, or identify variability, are more likely to have extreme values. For the metrics activity ratio (*activity_ratio*), rarity recording (*median_diff_rarity*), periodicity (*periodicity*) and periodicity variation (*periodicity_variation*) using a cut-off of 10 active days reduces the range of values produced by approximately 50%.

Additionally we tested the sensitivity of our findings to changes in the threshold used. We re-analysed our data using thresholds of 7 and 15 (approximately a third higher and lower than 10). We found that the results of the principal component and therefore the definition of our axes of recorder behaviour were unchanged.

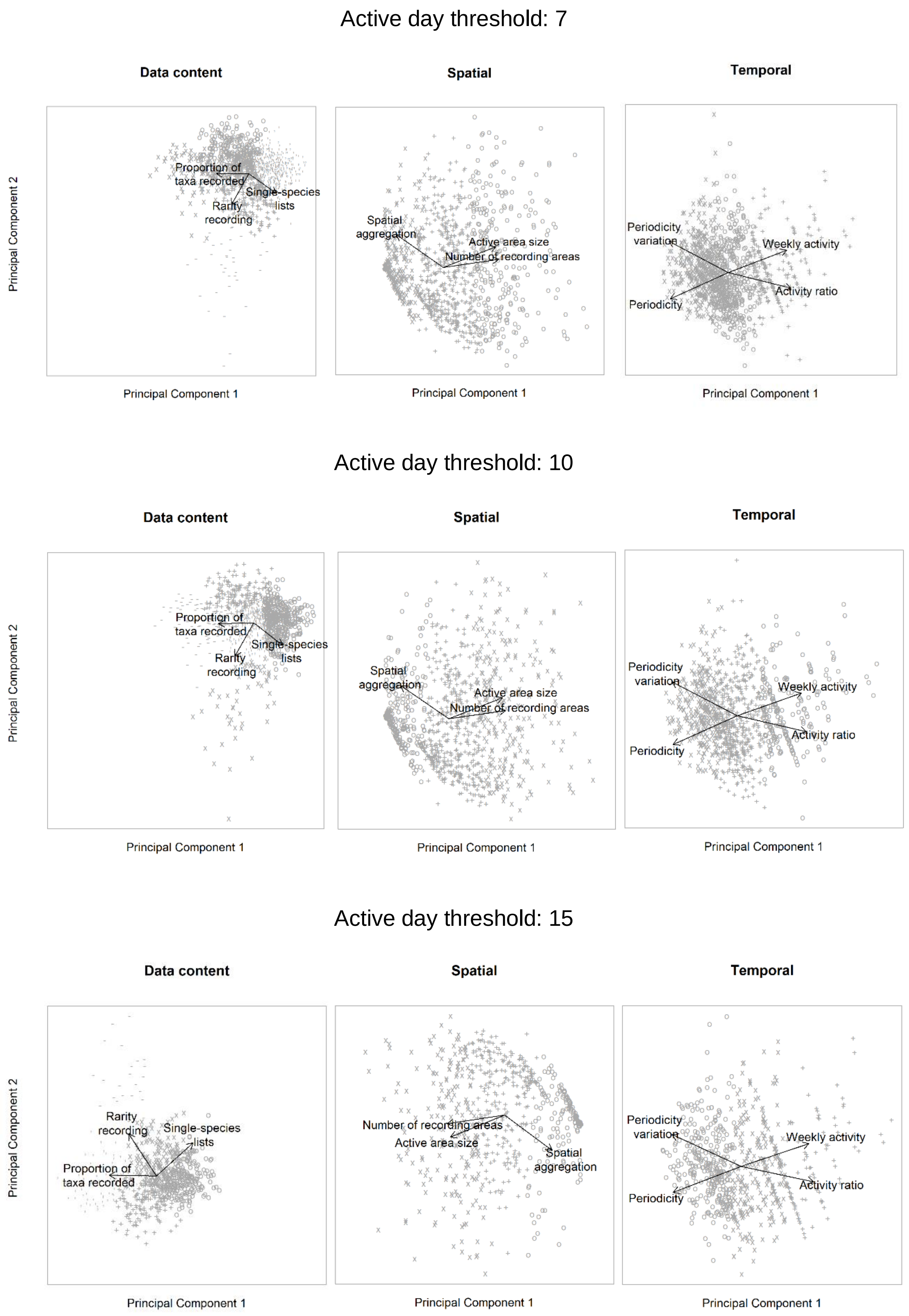
Active day threshold: 7
Data content
Spatial
Temporal
Principal Component 2
Proportion of taxa recorded
Single-species lists
Rarity recording
Spatial aggregation
Active area size
Number of recording areas
Periodicity variation
Weekly activity
Activity ratio
Periodicity
Principal Component 1
Active day threshold: 10
Data content
Spatial
Temporal
Principal Component 2
Proportion of taxa recorded
Single-species lists
Rarity recording
Spatial aggregation
Active area size
Number of recording areas
Periodicity variation
Weekly activity
Activity ratio
Periodicity
Principal Component 1
Active day threshold: 15
Data content
Spatial
Temporal
Principal Component 2
Rarity recording
Single-species lists
Proportion of taxa recorded
Number of recording areas
Active area size
Spatial aggregation
Periodicity variation
Weekly activity
Activity ratio
Periodicity
Principal Component 1

Figure S2 The results of principal components analysis using a threshold number of active days of 7, 10 and 15. Principal components analysis undertaken using the three sets of participant metrics: temporal, spatial and data content. Symbols represent the clusters identified via k-means clustering, but which have low support across all analyses. Note that the results for each threshold are essentially the same. Though the PCA axes in some cases are reversed this does not affect the interpretation of the results. The figures used under the heading of the 10 active day threshold are the same as those used in figure 3 in the manuscript.

2 – Principal components analysis of all metrics in a single analysis

Here we present the results of a principal components analysis of all metrics (temporal, spatial and data content), together in a single analysis. This used the same approach as described in the manuscript, other than the combination of all metrics in a single analysis.
The top four axes in this combine analysis describe 82% of all the variation (table S1).

**Component**	**1**	**2**	**3**	**4**
**Standard Deviation**	1.8809	1.5726	1.1119	0.97656
**Proportion of variance**	0.3538	0.2473	0.1236	0.09537
**Cumulative proportion of variance**	0.3538	0.6011	0.7247	0.82010

Table S1 – The results of a principal components analysis of all temporal, spatial and data content metrics.
Since this PCA contains 10 metrics it can be hard to create a clear interpretation of the meaning of each of the principal components (figure S1). To aid comparison we examined the four principal components that describe the greatest variation with the four axes presented in the main body of the manuscript. If the same four interpretations for these four axes are found in both analyses it supports the conclusion that our choice to analyse the three sets of metrics in isolation did not affect the outcome of the analysis.

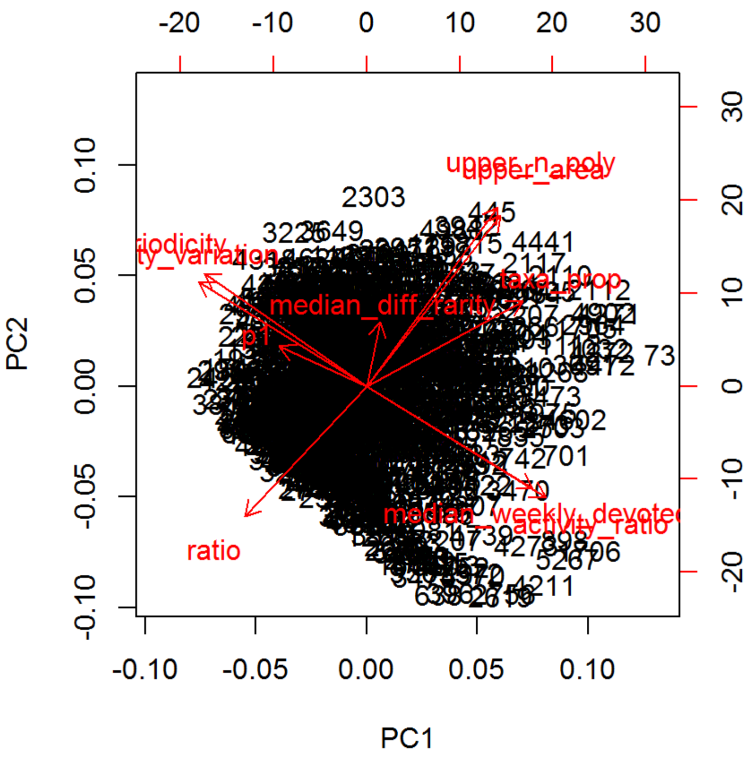

Figure S3 – Biplot showing the relative importance of each metric on the first two principal components
We present the loadings of the combined PCA in table S2. To aid interpretation we have shown in bold the loading values with an absolute value greater than 0.35.

Component	PC1	PC2	PC3	PC4
**Activity ratio**	**0.404**	-0.300	0.081	-0.134
**Weekly activity**	**0.375**	-0.278	0.108	-0.104
**Periodicity**	**-0.366**	0.305	-0.078	0.015
**Periodicity variation**	**-0.380**	0.281	-0.056	0.189
**Active area size**	0.302	**0.460**	0.077	-0.021
**Number of recording areas**	0.297	**0.482**	0.172	0.031
**Spatial aggregation**	-0.275	**-0.352**	-0.329	-0.102
**Proportion of Taxa Recorded**	**0.352**	0.229	**-0.457**	0.106
**Rarity Recording**	0.030	0.173	**-0.553**	**-0.719**
**Single-species lists**	-0.197	0.107	**0.561**	**-0.629**

Table S2 – The loadings of each metric in the combined PCA. The loadings with absolute values greater than 0.35 are highlighted in bold. These are the most important metrics for driving each principal component and can be used to interpret the meaning of the axis.

The first principal component is positively related to activity ratio, weekly activity, and the proportion of taxa recorded, and negatively related to periodicity and periodicity variation. This is very similar to the recording intensity axes identified in the PCA of temporal metrics (figure 3a), but with the addition of the data content metric 'proportion of taxa recorded'.

The second principal component is driven by the three spatial metrics in the same configuration as in the PCA of spatial metrics in isolation, meaning that this can be interpreted as spatial extent in the same way as in the main body of the paper (figure 3b)

The third principal component features all three of the data content metrics and the pattern of their loading is the same to the recording potential axis in the data-content-only PCA (figure 3c).

The fourth axis is very strongly driven by only two data content metrics; rarity recording and single-species lists. This is the same as the result for the second principal component in the data-content-only analysis where, as here, proportion of taxa recorded has very little effect. The fourth axis in the combined analysis can there for be interpreted as rarity recording, as it is in the data-content-only analyses (figure 3c).

All four axes in this combined analysis can be mapped clearly onto the four axes identified in the PCAs of each set of metrics in isolation. The drawback of this combined approach is that some principal components are being driven, by a lesser extent, by the other metrics. For example, in this combined analysis the first principal component has seven metrics with absolute loading values between 0.3 and 0.4. While the interpretation is similar to that in the temporal-metrics-only PCA presented in the body of the paper, the addition of other metrics with small, but not insignificant loading values, makes the interpretation less clear-cut. When using an absolute loading value cut-off of 0.35, only one metric is found in a principal component with metrics from another set (proportion of taxa recorded [data content metric] in principal component one [temporal metrics]). This generally supports our *a prori* hypothesis that metrics in one group (temporal, spatial, or data content) were not dependent on any other group.

3 – Estimating recorder metrics for spatial subsets

The metrics presented in main manuscript use all data available for their estimation. There are cases however where sub-setting the data prior to the estimation of metrics could be warranted. For example if you are calculating metrics for a citizen science project that spans Europe or the India then the species composition will vary significantly spatially and recorders are unlikely to have the ability to sample in all locations. As a consequence the rarity recording metric and the proportion of taxa recorded metric might not be suitable for your purpose.

To account for this we suggest spatial sub-setting and outline how this can be done here. In the case of Europe, we might decide to subset by country, since this will be the primary barrier to movement. In India where barriers are less discrete we might instead use buffers. We will demonstrate both methods here using our case study data from the UK.

Methods

The methods are straight forward. We simply subset the data prior to calculating metrics. In the case of country estimates we subset the data by country and then estimate the metrics for all the users with records in that country. If a recorder records in more than one country you will have estimates for each country they have recorded in. Using a buffer we draw a line a set distance around a recorders observations and use this to select data from all recorders that fall within the buffer and use this data to estimate the metrics

By Country

Here we will calculate the rarity recording metric by country for our example data.

```
library(recorderMetrics, quietly = TRUE)

## Registered S3 methods overwritten by 'adehabitatMA':
##   method                       from
##   print.SpatialPixelsDataFrame sp
##   print.SpatialPixels          sp

# load example data
head(cit_sci_data)

##        recorder species       date       long      lat  km_sq
## 82138     11652       1 2017-08-19 -2.4872953 51.31455 ST6657
## 150797     3007      52 2014-06-12 -2.2452737 50.62048 SY8280
## 80713     22725      43 2015-07-05 -1.9939669 50.64902 SZ0083
## 161905    22725      26 2017-04-18 -2.4787477 50.81198 ST6601
## 217        1417       2 2017-03-31 -1.3645562 53.51558 SE4202
## 134124    26865      56 2017-08-05 -0.1137257 50.90479 TQ3213

library(sp)

## Warning: package 'sp' was built under R version 3.6.3

plot(GB)
```

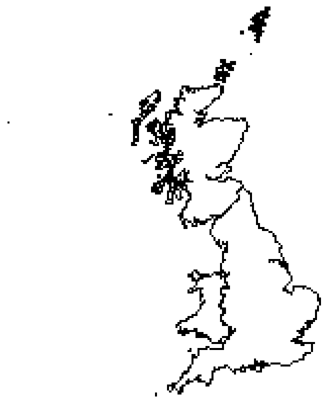

I have downloaded the shape files for the countries that make up Great Britain and will be using these for sub-setting. You can download similar boundaries from here: https://www.naturalearthdata.com/downloads/10m-cultural-vectors/

Next we convert our data into a spatial object.

```
# Convert our citizen science data to a SpatialPointsDataframe
SP <- SpatialPointsDataFrame(data = cit_sci_data,
                             coords = cit_sci_data[,c('long','lat')])
# Define lat long coordinate system
CRS("+proj=longlat +ellps=WGS84 +datum=WGS84 +no_defs")

## CRS arguments:
##  +proj=longlat +ellps=WGS84 +datum=WGS84 +no_defs +towgs84=0,0,0

proj4string(SP) <- CRS("+proj=longlat +ellps=WGS84 +datum=WGS84 +no_defs")

plot(SP)
```

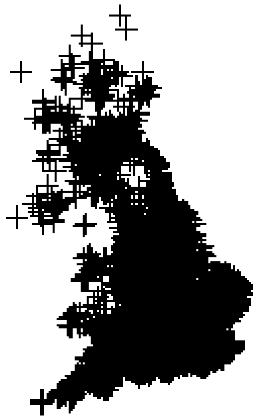

Now we loop though each country in my shape file (England, Scotland, Wales), and calculate the metrics for all recorders with records in those countries

```
# Empty object for all results
SR_all_countries <- NULL

# Loop through counties
for(i in unique(GB$NAME)){

  # Subset by country
  SP_C <- SP[GB[GB$NAME == i, ], ]

  # Calculate the metric within country
  SR_one_country <- lapply(unique(SP_C$recorder),
                           FUN = speciesRarity,
                           data = SP_C@data,
                           sp_col = 'species',
                           recorder_col = 'recorder')

  # combine data
  SR_one_country <- do.call(rbind, SR_one_country)
  SR_one_country$country <- i
  SR_all_countries <- rbind(SR_all_countries,
                            SR_one_country)
}

# How many recorders are found in more than one country
sum(table(SR_all_countries$recorder) > 1)

## [1] 75
```

```r
# Store their IDs
IDs <- names(table(SR_all_countries$recorder)[table(SR_all_countries$recor
der) > 1])
```

It is important to note that in this data set there are 75 recorders who have recorded in more than one country and as a result they appear more than once in our results.

When we plot the data for the country estimates versus the global estimates we can see the difference this makes.

```r
# Run the metric for all recorders globally
SR_all <- lapply(unique(cit_sci_data$recorder),
                 FUN = speciesRarity,
                 data = cit_sci_data,
                 sp_col = 'species',
                 recorder_col = 'recorder')

# summarise as one table
SR_all_sum <- do.call(rbind, SR_all)

# Compare results with original analysis
combo <- merge(y = SR_all_countries,
               x = SR_all_sum,
               by = 'recorder')

# I'm removing people who have recorded in more than one
# country and those with fewer than 10 records
plot(combo$median_diff_rarity.x[combo$n.x > 10 & !combo$recorder %in% IDs]
,
     combo$median_diff_rarity.y[combo$n.x > 10 & !combo$recorder %in% IDs]
,
     xlab = 'Original',
     ylab = 'By Country',
     main = 'Median Rarity Difference')
abline(0,1)
```

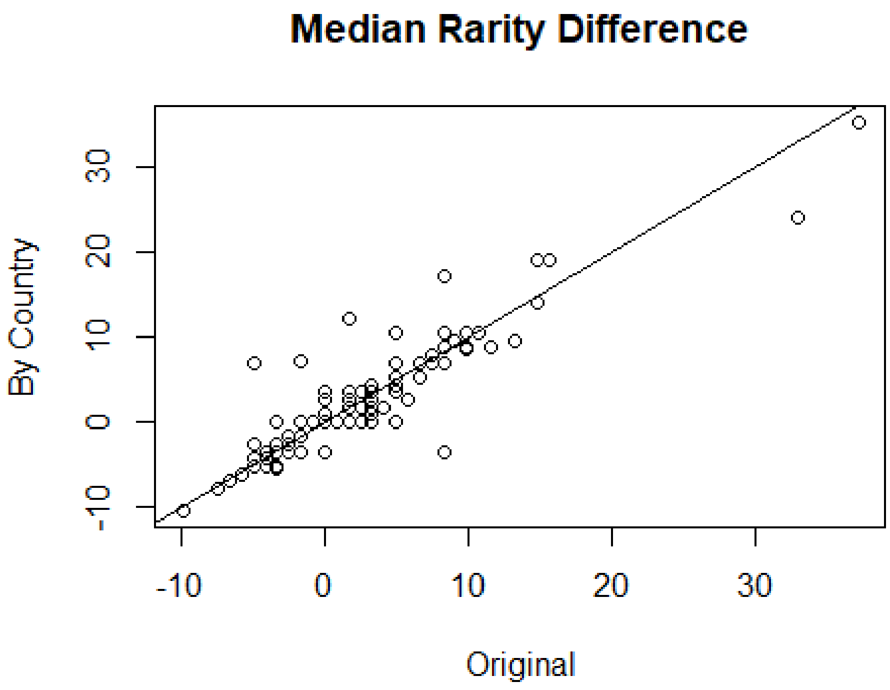

For a small number of recorders this makes a significant difference to their estimates.

By Buffer

Here we will calculate the proportion of taxa recorded metric using buffers around each recorder. Note that we have already converted our data to be spatial in the example above so I won't repeat that here.

Here I use a buffer size of 30km.

```
library(raster)

## Warning: package 'raster' was built under R version 3.6.2

library(rgeos)

## Warning: package 'rgeos' was built under R version 3.6.3

## rgeos version: 0.5-2, (SVN revision 621)
##  GEOS runtime version: 3.6.1-CAPI-1.10.1
##  Linking to sp version: 1.4-1
##  Polygon checking: TRUE

# Empty object for all results
TB_all_buffers <- NULL

for(i in unique(SP$recorder)){

  SP_R <- SP[SP$recorder == i, ]
  SP_R_buffer <- buffer(SP_R, 30000)
  SP_P <- SP[SP_R_buffer, ]

  TB_one_buffer <- taxaBreadth(recorder_name = i,
```

```
                                              data = SP_P@data,
                                              sp_col = 'species',
                                              recorder_col = 'recorder')

  TB_all_buffers <- rbind(TB_all_buffers,
                          TB_one_buffer)
}

# Compare results with original global analysis
TB_all <- lapply(unique(cit_sci_data$recorder),
                 FUN = taxaBreadth,
                 data = cit_sci_data,
                 sp_col = 'species',
                 recorder_col = 'recorder')

# summarise as one table
TB_all_sum <- do.call(rbind, TB_all)

# combine the original and buffer data
combo <- merge(y = TB_all_buffers,
               x = TB_all_sum,
               by = 'recorder')

plot(combo$taxa_prop.x[combo$n.x > 10],
     combo$taxa_prop.y[combo$n.x > 10],
     xlab = 'Original',
     ylab = 'By buffer',
     main = 'Proportion of taxa recorded')
abline(0,1)
```

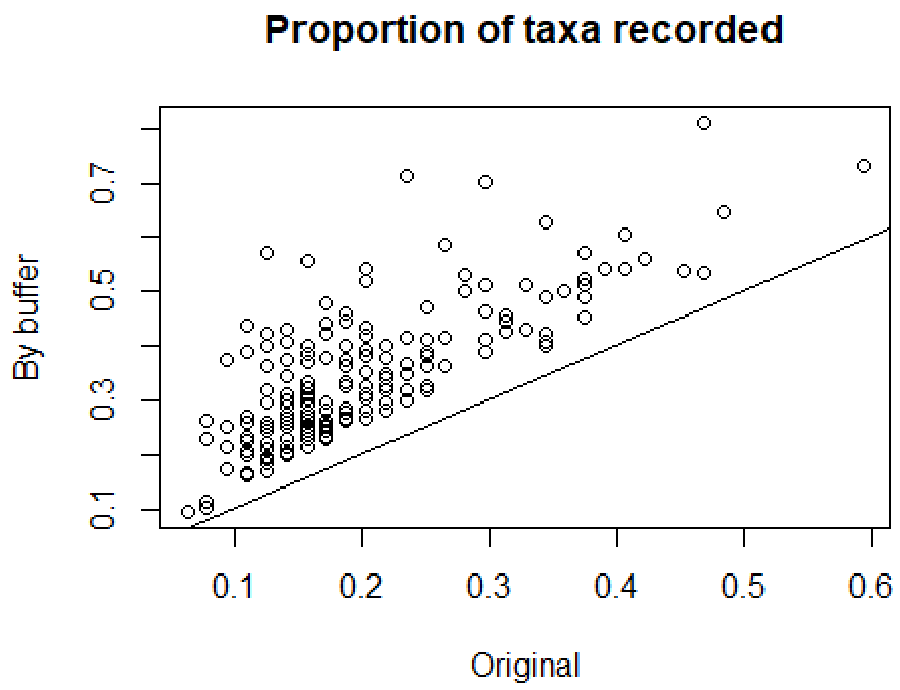

The size of the buffer is very important and if you were to use this method we strongly suggest that you examine carefully the size of buffer you are going to use. If the buffer is too large then you will not account for the spatial variation that you are trying to account for. If the buffer is too small then there will be little other data within the buffer resulting in estimates that are primarily influenced by the recorders observations.

To demonstrate this we re-ran this analysis using 5 different buffer sizes

```
# Test a number of different buffer sizes (km)
buffer_sizes <- c(5, 10, 30, 50, 100, 200)

for(buffer_size in buffer_sizes){

  TB_all_buffers <- NULL

  for(i in unique(SP$recorder)){

    SP_R <- SP[SP$recorder == i, ]
    SP_R_buffer <- buffer(SP_R, buffer_size * 1000)
    SP_P <- SP[SP_R_buffer, ]

    TB_one_buffer <- taxaBreadth(recorder_name = i,
                                 data = SP_P@data,
                                 sp_col = 'species',
                                 recorder_col = 'recorder')

    TB_all_buffers <- rbind(TB_all_buffers,
                            TB_one_buffer)
  }

  # combine the original and buffer data
  combo <- merge(y = TB_all_buffers,
                 x = TB_all_sum,
                 by = 'recorder')

  plot(combo$taxa_prop.x[combo$n.x > 10],
       combo$taxa_prop.y[combo$n.x > 10],
       xlab = 'Original',
       ylab = 'By buffer',
       main = paste('Proportion of taxa recorded - Buffer',
                    paste0(buffer_size, 'km')))
  abline(0,1)
}
```

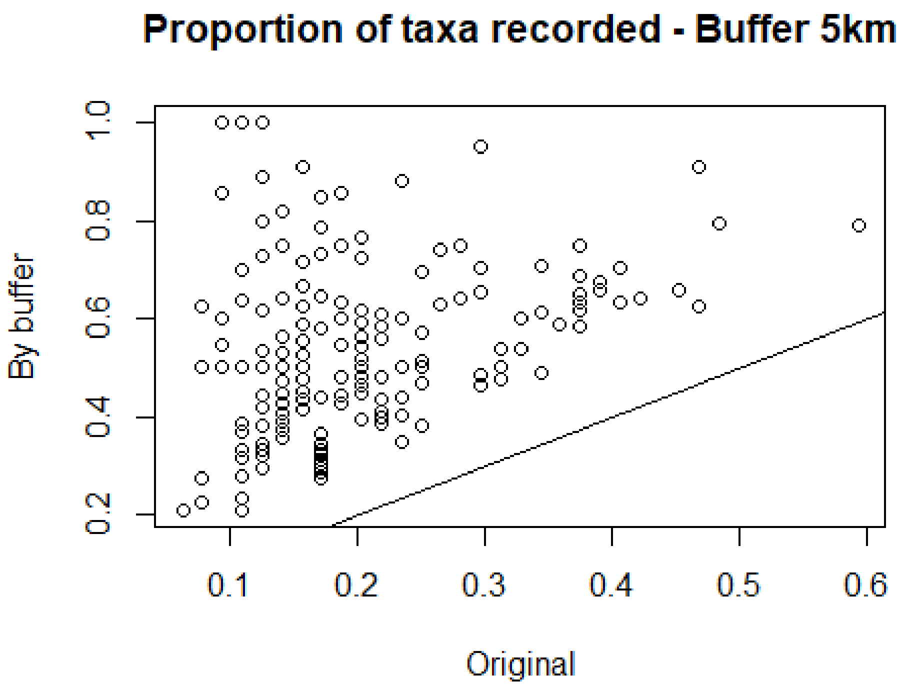
Proportion of taxa recorded - Buffer 5km
By buffer
0.2
0.4
0.6
0.8
1.0
0.1
0.2
0.3
0.4
0.5
0.6
Original

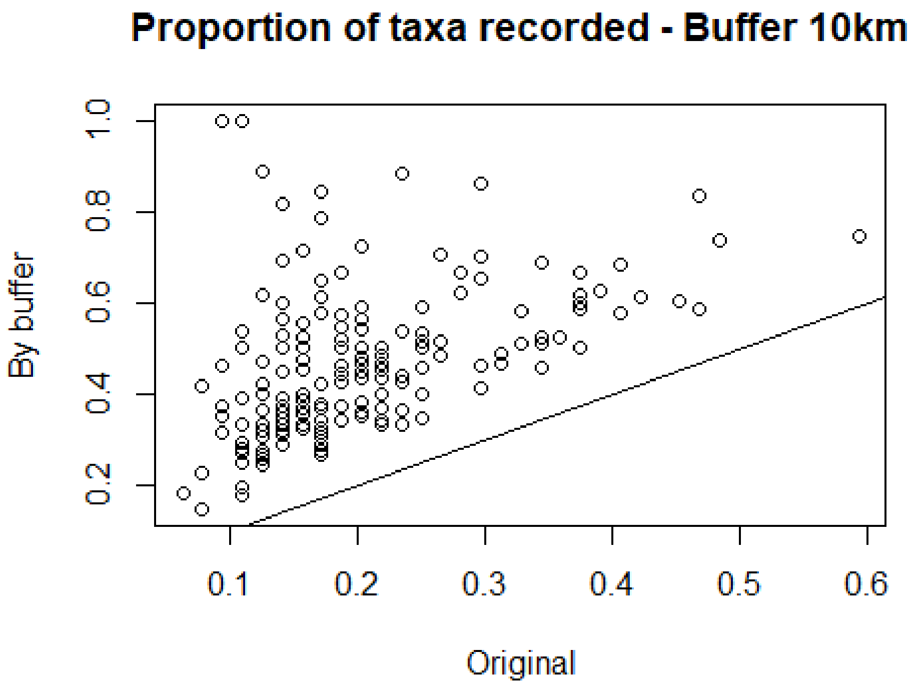
Proportion of taxa recorded - Buffer 10km
By buffer
0.2
0.4
0.6
0.8
1.0
0.1
0.2
0.3
0.4
0.5
0.6
Original

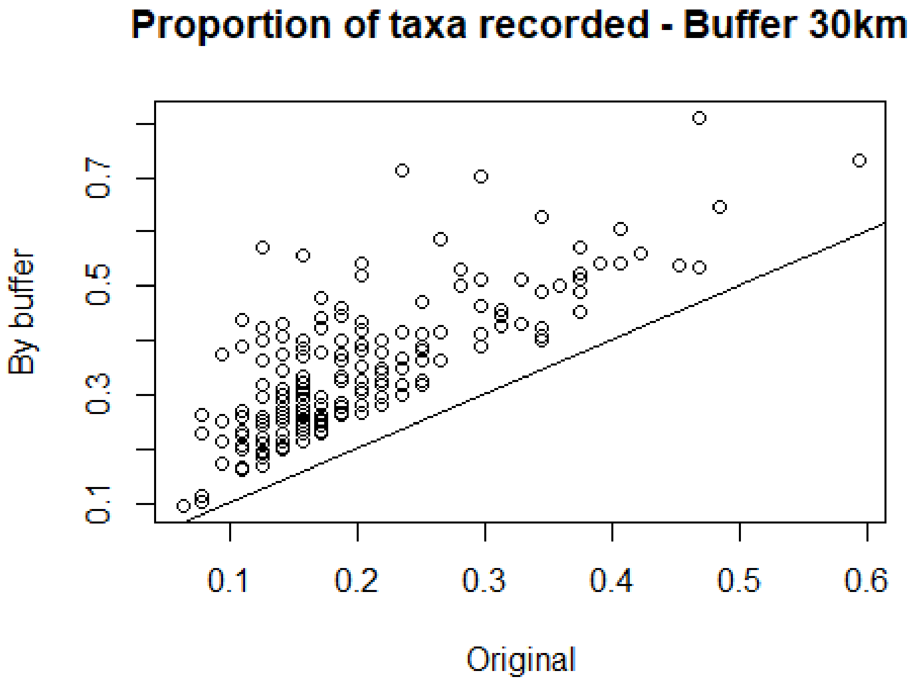
Proportion of taxa recorded - Buffer 30km
By buffer
0.1
0.3
0.5
0.7
0.1
0.2
0.3
0.4
0.5
0.6
Original

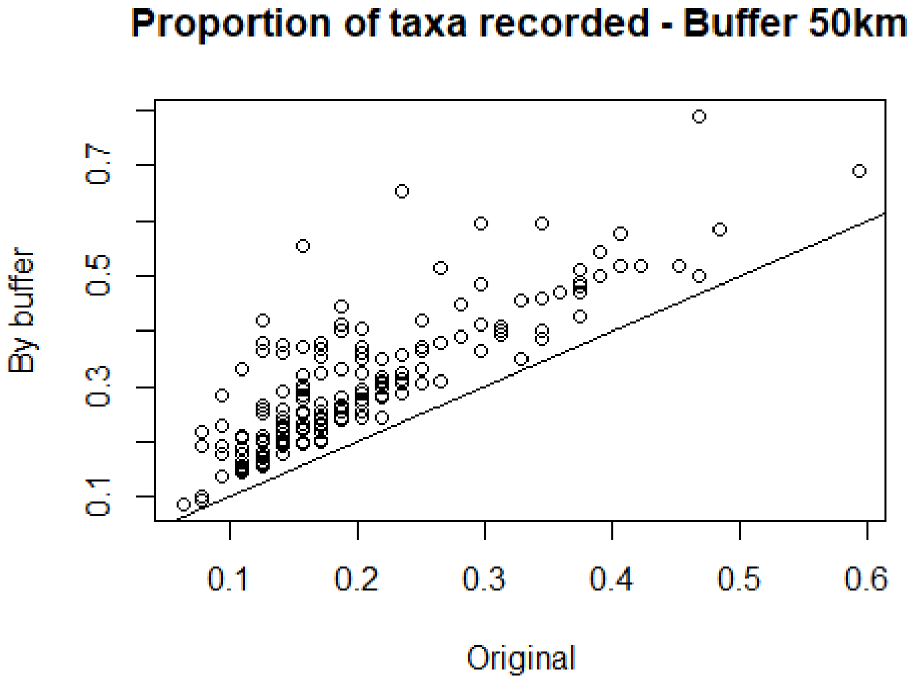
Proportion of taxa recorded - Buffer 50km
By buffer
0.1
0.3
0.5
0.7
0.1
0.2
0.3
0.4
0.5
0.6
Original

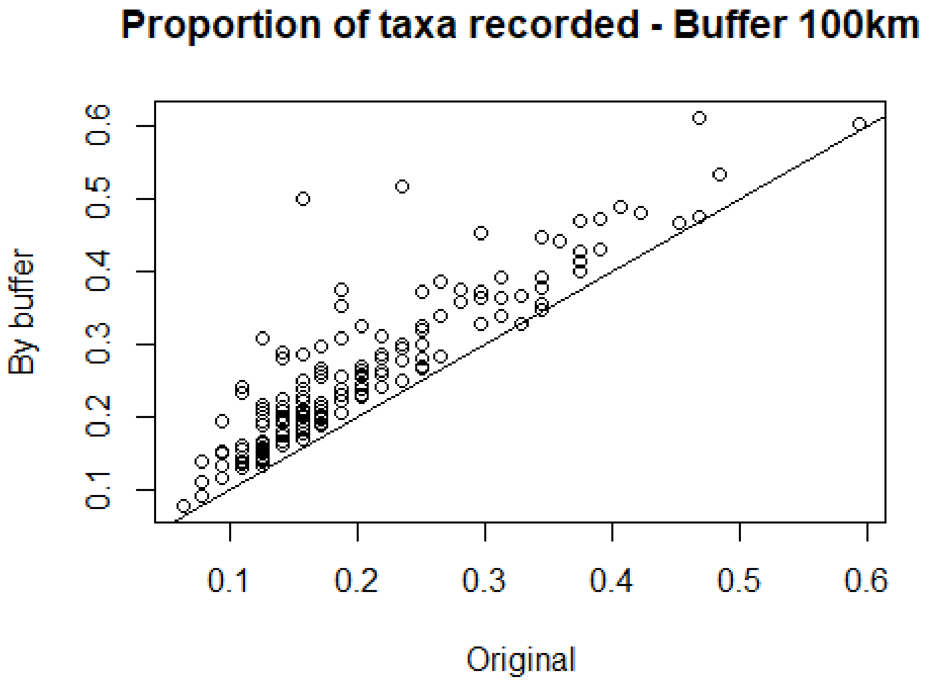
Proportion of taxa recorded - Buffer 100km
By buffer
0.1
0.2
0.3
0.4
0.5
0.6
0.1
0.2
0.3
0.4
0.5
0.6
Original

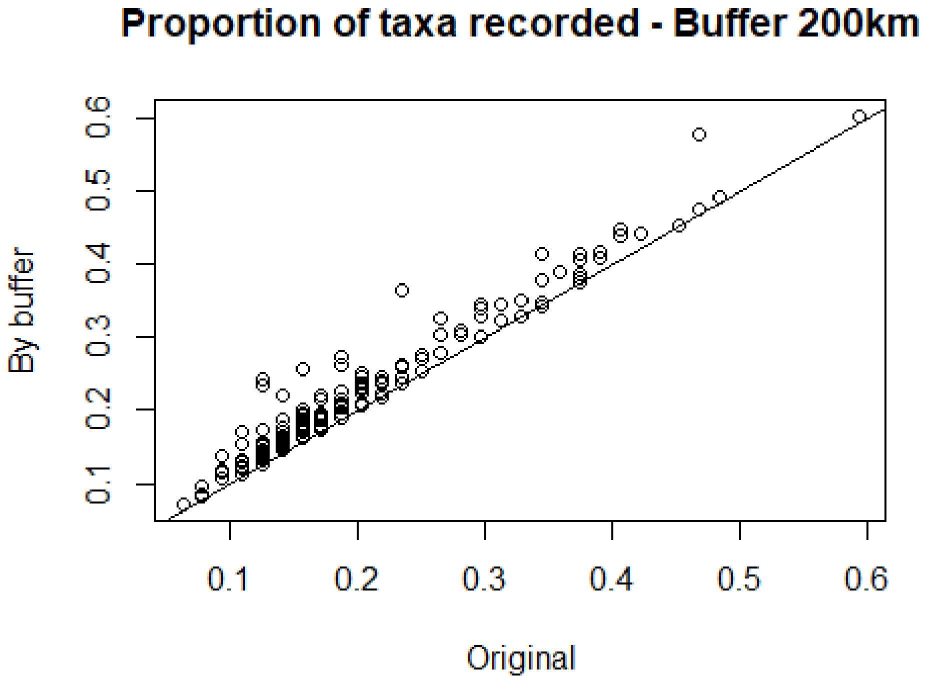
Proportion of taxa recorded - Buffer 200km
By buffer
0.1
0.2
0.3
0.4
0.5
0.6
0.1
0.2
0.3
0.4
0.5
0.6
Original
